# Exploring Type-Level Bisimilarity towards More Expressive Multiparty Session Types

**DOI:** 10.1007/978-3-030-44914-8_10

**Published:** 2020-04-18

**Authors:** Sung-Shik Jongmans, Nobuko Yoshida

**Affiliations:** ETH Zurich, Zurich, Switzerland; 9 Department of Computer Science, Open University, Heerlen, the Netherlands; 10 CWI, Amsterdam, the Netherlands; 11 Department of Computing, Imperial College, London, UK

## Abstract

A key open problem with multiparty session types (MPST) concerns their expressiveness: current MPST have inflexible choice, no existential quantification over participants, and limited parallel composition. This precludes many real protocols to be represented by MPST. To overcome these bottlenecks of MPST, we explore a new technique using weak bisimilarity between global types and endpoint types, which guarantees deadlock-freedom and absence of protocol violations. Based on a process algebraic framework, we present well-formed conditions for global types that guarantee weak bisimilarity between a global type and its endpoint types and prove their check is decidable. Our main practical result, obtained through benchmarks, is that our well-formedness conditions can be checked orders of magnitude faster than directly checking weak bisimilarity using a state-of-the-art model checker.

### 1 Introduction

**Background.** To take advantage of modern parallel and distributed computing platforms, message-passing concurrency is becoming increasingly important. Modern programming languages, however, offer insufficiently effective linguistic support to guide programmers towards *safe usage* of message-passing abstractions (e.g., to prevent deadlocks or protocol violations).

*Multiparty session types* (MPST) [34] constitute a static, correct-by-construction approach to simplify concurrent programming, by offering a type-based framework to specify message-passing protocols and ensure deadlock-freedom and protocol conformance. The idea is to use behavioural types [1,37] to enforce *protocols* (i.e., patterns of admissible communications) between *roles* (e.g., threads, processes, services) to avoid concurrency bugs. The framework is illustrated in Fig. 1: first, a *global type* $G$ (protocol specification; written by the programmer) is *projected* onto every role; then, every resulting *endpoint type (local type)* $L_i$ (role specification) is *type-checked*

Fig. 1: MPST framework with the corresponding *process* $P_i$ (role implementation). If every process is well-typed against its local type, then their parallel composition is guaranteed to be free of deadlocks and protocol violations relative to the global type. Notably, common concurrency bugs as sends without receives, receives without sends, and type mismatches (actual type sent vs. expected type received) are ruled out statically. The MPST framework is *language-agnostic*: in recent years, practical implementations of MPST have been developed for several programming languages, including Erlang, F#, Go, Java, and Scala [18,35,36,45,46,50].

**Three open problems.** Many practically relevant protocols *cannot* be specified as global types; this limits MPST's applicability to real-world concurrent programs. Specifically, while the original work [33] has been extended with several advanced features (e.g., time [7,44], security [11,12,13,17], and parametrisation [18,25,47]), core features still have significant restrictions: inflexible choice, no existential quantification over participants, and limited parallel composition.

**1. Inflexible choice:** In the original work [33], if there is a choice between multiple branches, the sender in the first communication of each branch must be the same, the receiver must be the same, and the message type must be different (i.e., no non-determinism). Moreover, each role *not* involved in the first communication of each branch, must have the same behaviour in each continuation. For instance, the following global type specifies a protocol where Client $\mathsf{c}$ repeatedly requests an arithmetic Server $\mathsf{s}$ to compute the sum or product of two numbers:

$$\mu X.\left[\left[\mathsf{c}\twoheadrightarrow\mathsf{s}:\mathsf{Add}\cdot\mathsf{s}\twoheadrightarrow\mathsf{c}:\mathsf{Sum}\cdot X\right]+\left[\mathsf{c}\twoheadrightarrow\mathsf{s}:\mathsf{Mul}\cdot\mathsf{s}\twoheadrightarrow\mathsf{c}:\mathsf{Prod}\cdot X\right]\right]$$

Here, $\mathsf{c}\twoheadrightarrow\mathsf{s}:\mathsf{Add}$ specifies a communication of an $\mathsf{Add}$-message (with two numbers as payload) from the Client to the Server, while $\cdot$ and $+$ specify sequencing and branching, and square brackets indicate operator precedence. This is a "good" global type that satisfies the conditions. In contrast, the following "bad" global type specifies a protocol where Client $\mathsf{c}$ repeatedly requests addition and multiplication Servers $\mathsf{s}_1$ and $\mathsf{s}_2$ via Router $\mathsf{r}$ (payload types omitted; $r_1\twoheadrightarrow r_2\twoheadrightarrow r_3:t$ abbreviates $r_1\twoheadrightarrow r_2:t\cdot r_2\twoheadrightarrow r_3:t$):

$$\mu X.\left[\left[\mathsf{c}\twoheadrightarrow\mathsf{r}\twoheadrightarrow\mathsf{s}_1:\mathsf{Add}\cdot\mathsf{s}_1\twoheadrightarrow\mathsf{c}:\mathsf{Sum}\cdot X\right]+\left[\mathsf{c}\twoheadrightarrow\mathsf{r}\twoheadrightarrow\mathsf{s}_2:\mathsf{Mul}\cdot\mathsf{s}_2\twoheadrightarrow\mathsf{c}:\mathsf{Prod}\cdot X\right]\right]$$

Several improvements to the original work have been proposed: Honda et al. managed to allow each role $r$ not involved in a choice to have different behaviour in different branches [15], so long as $r$ is made aware of which branch is chosen in a timely and unambiguous fashion (e.g., the previous global type is still forbidden), while Lange et al., Castagna et al., and Hu & Yoshida managed to allow choices between different receivers [16,23,36,40]. For instance, the following global type (the Client *directly* requests the specialised server) is allowed:

$$\mu X.\left[\left[\mathsf{c}\twoheadrightarrow\mathsf{s}_1:\mathsf{Add}\cdot\mathsf{s}_1\twoheadrightarrow\mathsf{c}:\mathsf{Sum}\cdot X\right]+\left[\mathsf{c}\twoheadrightarrow\mathsf{s}_2:\mathsf{Mul}\cdot\mathsf{s}_2\twoheadrightarrow\mathsf{c}:\mathsf{Prod}\cdot X\right]\right]$$

But, the following global type (two Clients $\mathsf{c}_1$ and $\mathsf{c}_2$ use Server $\mathsf{S}$) is forbidden:

$$\mu X.\begin{bmatrix}\left[\mathsf{c}_1\twoheadrightarrow\mathsf{s}:\mathsf{Add}\cdot\mathsf{s}\twoheadrightarrow\mathsf{c}_1:\mathsf{Sum}\cdot X\right]+\left[\mathsf{c}_1\twoheadrightarrow\mathsf{s}:\mathsf{Mul}\cdot\mathsf{s}\twoheadrightarrow\mathsf{c}_1:\mathsf{Prod}\cdot X\right]+\\ \left[\mathsf{c}_2\twoheadrightarrow\mathsf{s}:\mathsf{Add}\cdot\mathsf{s}\twoheadrightarrow\mathsf{c}_2:\mathsf{Sum}\cdot X\right]+\left[\mathsf{c}_2\twoheadrightarrow\mathsf{s}:\mathsf{Mul}\cdot\mathsf{s}\twoheadrightarrow\mathsf{c}_2:\mathsf{Prod}\cdot X\right]\end{bmatrix}$$

None of the existing works allow the above nondeterministic choices between different senders. We call this the **+-problem**: how to add a choice constructor, denoted by +, to specify choices between disjoint sender-receiver-label triples?

**2. No existential quantification:** Related to the +-problem is the **∃-problem**: how to add an existential role quantifier, denoted by ∃, to specify the execution of ∃'s body for some role in ∃'s domain? For instance, instead of writing a separate global type for 2 Clients, 3 Clients, etc., existential role quantification allows us to write *only one* global type for any $n{>}1$ Clients:

$$\mu X.\, \exists r{\in}\{\mathsf{c}_i \mid 1{\leq}i{\leq}n\}.\, \big[\big[r \twoheadrightarrow \mathsf{s}{:}\mathsf{Add} \cdot \mathsf{s} \twoheadrightarrow r{:}\mathsf{Sum} \cdot X\big] + \big[r \twoheadrightarrow \mathsf{s}{:}\mathsf{Mul} \cdot \mathsf{s} \twoheadrightarrow r{:}\mathsf{Prod} \cdot X\big]\big]$$

The ∃-problem was first formulated by Deniélou & Yoshida [22] as the dual of the ∀-problem (i.e., specify the execution of ∀'s body for each role in ∀'s domain): the ∀-problem was solved in the same paper, but the ∃-problem "raises many semantic issues" [22] and has remained open for almost a decade.

**3. Limited parallel composition:** The third open problem related to choice is the **‖-problem**: how to add a constructor, denoted by ‖, that allows *infinite branching* (i.e., non-finite control) through unbounded parallel interleaving? While extensions of the original work with parallel composition exist (e.g., [16,22,23,43]), none of these works supports unbounded interleaving. For instance, the following global type allows an unbounded number of requests to be served by the Server in parallel (instead of sequentializing them):

$$\mu X.\, \exists r{\in}\{\mathsf{c}_i \mid 1{\leq}i{\leq}n\}.\, \big[\big[r \twoheadrightarrow \mathsf{s}{:}\mathsf{Add} \cdot \big[\mathsf{s} \twoheadrightarrow r{:}\mathsf{Sum} \parallel X\big]\big] + \big[r \twoheadrightarrow \mathsf{s}{:}\mathsf{Mul} \cdot \big[\mathsf{s} \twoheadrightarrow r{:}\mathsf{Prod} \parallel X\big]\big]\big]$$

**Contributions.** We overcome these three bottlenecks of MPST with an approach based on three key novelties: first, we have a new definition of projection that keeps more information in the local types than existing definitions; second, we exploit this extra information to formulate our well-formedness conditions; third, we use an unexplored proof method for MPST, namely to prove the operational equivalence between a global type and its projections modulo weak bisimilarity. This makes the proofs cleaner and ultimately allows for more flexibility (e.g., our approach can be modularly combined with traditional session type checking, but potentially also with other verification methods, such as model checking or conformance testing). To summarise the highlights:

– For the first time, we provide solutions to the +-problem, the ∃-problem, and the ‖-problem, by presenting expressive syntax for global and local types (formulated as process algebraic terms), a refined notion of projection, and novel well-formedness conditions.
– Our main theoretical result is *operational equivalence*: a well-formed global type behaves the same as the parallel composition of its projections, modulo weak bisimulation. This implies freedom of deadlocks and freedom of protocol violations of the projections. Checking this equivalence is decidable.
  To our knowledge, we are the first to use (weak) bisimilarity to prove the correctness of a projection operator from global to local types. By doing so,

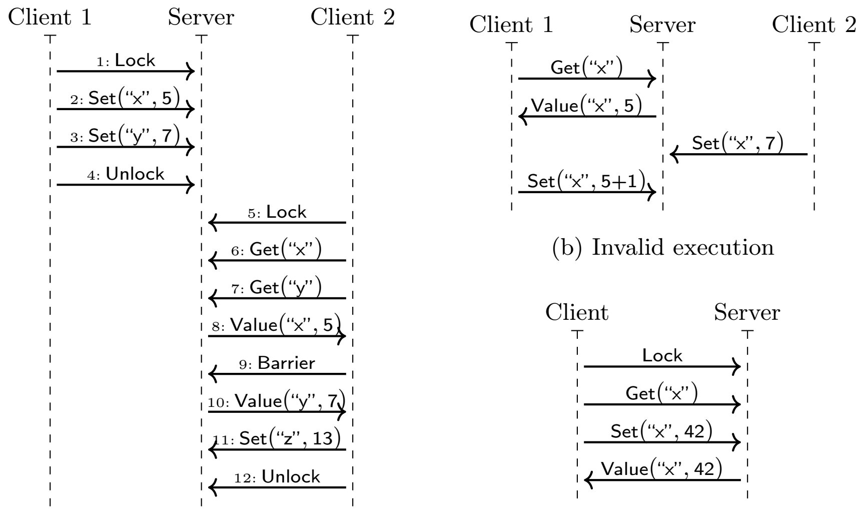

(a) Valid execution

(b) Invalid execution

(c) Invalid execution

Fig. 2: Example executions of the Key-Value Store protocol we decouple (a) the act of reasoning about projection and (b) the act of establishing compliance between local types and process implementations; until our work, these two concerns have always been conflated.
- Our main practical results are: (1) to provide representative protocols typable in our approach; and (2) the well-formedness conditions of (1) can be checked orders of magnitude faster than directly checking weak bisimilarity using mCRL2 [10,20,29], a state-of-the-art model checker.

In Sect. 2, we present an overview of our contribution through a representative example protocol that is not supported by previous work. In Sect. 3, we present the details of our theoretical contribution. In Sect. 4, we present the details of our practical contribution (implementation and evaluation). In Sect. 5, we discuss related work. We conclude and discuss future work in Sect. 6.

Detailed formal definitions and proofs of all lemmas and theorems can be found in our supplement [38].

### 2 Overview of our Approach

**Scenario.** To highlight our solutions to the +-problem, ∃-problem, and ‖-problem, we consider a *Key-Value Store* protocol, similar to those used in modern NoSQL databases [21,27]. Specifically, our Key-Value Store protocol is inspired by the transaction mechanism of the popular Redis database [48,49]. This protocol is not supported by any of the existing MPST works.

The Key-Value Store protocol consists of $n$ *Clients* that require access to the store, represented by role names $\mathsf{c}_1, ..., \mathsf{c}_n$, and one *Server* that provides access to the store, represented by role name $\mathsf{s}$. The store has keys of type $\mathsf{Str}$ (strings) and values of type $\mathsf{Nat}$ (numbers). Fig. 2 shows valid and invalid example executions of the protocol ($n$=2) as message sequence charts; it works as follows.

First, a $\mathsf{Lock}$-message is communicated from *some* Client $\mathsf{c}_i$ ($1 \leq i \leq n$) to Server $\mathsf{s}$ (Fig. 2a, arrows 1, 5); this grants $\mathsf{c}_i$ exclusive access to the store. Then, a sequence of messages to write and/or read values is communicated:

- To write, a $\mathsf{Set}$-message is communicated from $\mathsf{c}_i$ to $\mathsf{s}$ (arrows 2, 3, 11).
- To read, a $\mathsf{Get}$-message is communicated from $\mathsf{c}_i$ to $\mathsf{s}$ (arrows 6, 7). Then, *eventually*, a $\mathsf{Value}$-message is communicated from $\mathsf{s}$ to $\mathsf{c}_i$ (arrows 8, 10), but in the meantime, additional $\mathsf{Get}$-messages can be communicated from $\mathsf{c}_i$ to $\mathsf{s}$. In this way, the Client does not need to await the responses of the Server to perform multiple independent requests. To indicate enough $\mathsf{Get}$-messages have been sent, a $\mathsf{Barrier}$-message is communicated from $\mathsf{c}_i$ to $\mathsf{s}$ (arrow 9), which serves as a communication fence: the protocol will only proceed once all $\mathsf{Value}$-messages for pending $\mathsf{Get}$-messages have been communicated.

The sequence ends with the communication of an $\mathsf{Unlock}$-message from $\mathsf{c}_i$ to $\mathsf{s}$ (arrow 12). The protocol is then repeated for *some* Client $\mathsf{c}_j$ ($1 \leq j \leq n$); possibly, but not necessarily, $i$=$j$. In this way, the Server *atomically* processes accesses to the store between $\mathsf{Lock}$/$\mathsf{Unlock}$-messages.

**Global and local types.** The corresponding global type and local types, inferred via projection (for some $n$), are as follows:

$$G = \mu X.\, \exists r {\in} \{\mathsf{c}_i \mid 1 {\leq} i {\leq} n\}.\, r \twoheadrightarrow \mathsf{s} : \mathsf{Lock} \cdot \mu Y. \begin{bmatrix} \big[\mu Z.\, \big[\big[r \twoheadrightarrow \mathsf{s} : \mathsf{Get}(\mathsf{Str}) \cdot \big[\mathsf{s} \twoheadrightarrow r : \mathsf{Value}(\mathsf{Str}, \mathsf{Nat}) \parallel Z\big]\big] + r \twoheadrightarrow \mathsf{s} : \mathsf{Barrier}\big] \cdot Y\big] \\ + \big[r \twoheadrightarrow \mathsf{s} : \mathsf{Set}(\mathsf{Str}, \mathsf{Nat}) \cdot Y\big] + \big[r \twoheadrightarrow \mathsf{s} : \mathsf{Unlock} \cdot X\big] \end{bmatrix}$$

$$L_{\mathsf{c}_i} = \mu X.\, \mathsf{c}_i\mathsf{s}\,!\,\mathsf{Lock} \cdot \mu Y. \begin{bmatrix} \big[\mu Z.\, \big[\big[\mathsf{c}_i\mathsf{s}\,!\,\mathsf{Get}(\mathsf{Str}) \cdot \big[\mathsf{s}\mathsf{c}_i\,?\,\mathsf{Value}(\mathsf{Str}, \mathsf{Nat}) \parallel Z\big]\big] + \mathsf{c}_i\mathsf{s}\,!\,\mathsf{Barrier}\big] \cdot Y\big] \\ + \big[\mathsf{c}_i\mathsf{s}\,!\,\mathsf{Set}(\mathsf{Str}, \mathsf{Nat}) \cdot Y\big] + \big[\mathsf{c}_i\mathsf{s}\,!\,\mathsf{Unlock} \cdot X\big] \end{bmatrix}$$

$$L_{\mathsf{s}} = \mu X.\, \exists r {\in} \{\mathsf{c}_i \mid 1 \leq i \leq n\}.\, r\mathsf{s}\,?\,\mathsf{Lock} \cdot \mu Y. \begin{bmatrix} \big[\mu Z.\, \big[\big[r\mathsf{s}\,?\,\mathsf{Get}(\mathsf{Str}) \cdot \big[\mathsf{s}r\,!\,\mathsf{Value}(\mathsf{Str}, \mathsf{Nat}) \parallel Z\big]\big] + r\mathsf{s}\,?\,\mathsf{Barrier}\big] \cdot Y\big] \\ + \big[r\mathsf{s}\,?\,\mathsf{Set}(\mathsf{Str}, \mathsf{Nat}) \cdot Y\big] + \big[r\mathsf{s}\,?\,\mathsf{Unlock} \cdot X\big] \end{bmatrix}$$

Global type $r_1 \twoheadrightarrow r_2 : \ell(t)$ specifies the *communication* of a message labelled $\ell$ with a payload typed $t$ from sender $r_1$ to receiver $r_2$; global type $G_1 \cdot G_2$ specifies the *sequential composition* of global types $G_1$ and $G_2$; global type $G_1 + G_2$ specifies the *alternative composition* (choice) of global types $G_1$ and $G_2$; global type $\exists r {\in} \{r_1, ..., r_n\}.\, G$ specifies the *existential role quantification* over domain $\{r_1, ..., r_n\}$ (i.e., the alternative composition of $G[r_1/r]$ and ... and $G[r_n/r]$, where $G[r_i/r]$ denotes the substitution of $r_i$ for every $r$ in $G$); global type $G_1 \parallel G_2$ specifies the *interleaving composition* of $G_1$ and $G_2$ (free merge [4]); global type $\mu X.\, G$ specifies recursion (i.e., $X$ is bound to $\mu X.\, G$ in $G$).

Local type $r_1 r_2 ! \ell(t)$ specifies the send of a $\ell(t)$-message through the channel from $r_1$ to $r_2$; dually, local type $r_1 r_2 ? \ell(t)$ specifies a receive. Because every Client participates in only one branch of the quantification, their local types do not contain $\exists$ under the recursion. In contrast, because the Server participates in all branches, $L_{\mathsf{S}}$ does contain $\exists$ under the recursion.

By Thm. 3, $G$ and the parallel composition of $L_{\mathsf{C}_1}$, ..., $L_{\mathsf{C}_n}$, $L_{\mathsf{S}}$ are operationally equivalent (weakly bisimilar), which in turn implies deadlock-freedom and absence of protocol violations. Note also that our global type for the Key-Value Store protocol indeed relies on solutions to the +-problem (choice between multiple clients that send a $\mathsf{Lock}$-message), the $\exists$-problem (existential quantification over clients), and the $\|$-problem (unbounded interleaving to support asynchronous responses of a statically unknown number of requests).

### 3 An MPST Theory with $+$, $\exists$, and $\|$

#### 3.1 Types as Process Algebraic Terms

We define our languages of global and local types as *algebras* over sets of (global) communications and (local) sends/receives. This subsection presents preliminaries on the generic algebraic framework we use, based on the existing algebras PA [3] and TCP+REC [2]; the next subsection presents our specific instantiations for global and local types.

Let $\mathbb{A}$ denote a set of *actions*, ranged over by $\alpha$, and let $\{X_1, X_2, \ldots, Y, \ldots\}$ denote a set of *recursion variables*. Then, let $\text{Term}(\mathbb{A})$ denote the set of *(algebraic) terms*, ranged over by $T$, generated by the following grammar:

$$T ::= \boxed{1} \mid \alpha \mid T_1 + T_2 \mid T_1 \cdot T_2 \mid T_1 \| T_2 \mid X \mid \langle X_k \,|\, \{X_i \mapsto T_i\}_{i \in I} \rangle \;\; (k \in I)$$

Term $\boxed{1}$ specifies a *skip*; the grey background indicates it should not be explicitly written by programmers (but it is used only implicitly in the operational semantics). Term $\alpha$ specifies an atomic *action* from $\mathbb{A}$. Terms $T_1 + T_2$, $T_1 \cdot T_2$, and $T_1 \| T_2$ specify the *alternative composition*, the *sequential composition*, and the *interleaving composition* (free merge [4]; a form of parallel composition without interaction between the operands) of $T_1$ and $T_2$. Terms $X$ and $\langle X_k \,|\, \{X_i \mapsto T_i\}_{i \in I} \rangle$ specify *recursion*, where $\{X_i \mapsto T_i\}_{i \in I}$ is a *recursive specification* that maps recursion variables to terms, $X_k$ is the *initial call* (for $T_k$), and every $X_j$ that occurs in $T_k$ is a subsequent *recursive call* (for $T_j$); we write $\mu X. T$ instead of $\langle X \,|\, \{X \mapsto T\} \rangle$.

Let $\mathbb{X} \rightharpoonup \text{Term}(\mathbb{A})$ denote the set of all *recursive specifications* (i.e., every recursive specification is a partial function), ranged over by $E, F$, and let $\mathsf{sub}(E, T)$ denote the simultaneous substitution of term $E(X)$ for each recursion variable $X$ in $T$. Fig. 3 defines the operational semantics of terms. It consists of two components: relation $\rightarrow$ defines reduction of terms, while relation $\downarrow$ defines successful termination of terms. In words, term $T_1 + T_2$ is reduced by reducing either $T_1$ or $T_2$; term $T_1 \cdot T_2$ is reduced by reducing first $T_1$ and then $T_2$; term

$$\frac{}{\alpha \xrightarrow{\alpha} \boxed{1}} \quad \frac{T_1 \xrightarrow{\alpha} T_1'}{T_1 \cdot T_2 \xrightarrow{\alpha} T_1' \cdot T_2} \quad \frac{T_1\downarrow \quad T_2 \xrightarrow{\alpha} T_2'}{T_1 \cdot T_2 \xrightarrow{\alpha} T_2'} \quad \frac{T_1 \xrightarrow{\alpha} T_1'}{T_1 + T_2 \xrightarrow{\alpha} T_1'} \quad \frac{T_2 \xrightarrow{\alpha} T_2'}{T_1 + T_2 \xrightarrow{\alpha} T_2'}$$

$$\frac{T_1 \xrightarrow{\alpha} T_1'}{T_1 \parallel T_2 \xrightarrow{\alpha} T_1' \parallel T_2} \quad \frac{T_2 \xrightarrow{\alpha} T_2'}{T_1 \parallel T_2 \xrightarrow{\alpha} T_1 \parallel T_2'} \quad \frac{\mathsf{sub}(E, E(X)) \xrightarrow{\alpha} T'}{\langle X \,|\, E\rangle \xrightarrow{\alpha} T'}$$

(a) Reduction

$$\frac{}{\boxed{1}\downarrow} \quad \frac{T_1\downarrow}{T_1 + T_2\downarrow} \quad \frac{T_2\downarrow}{T_1 + T_2\downarrow} \quad \frac{T_1\downarrow \quad T_2\downarrow}{T_1 \cdot T_2\downarrow} \quad \frac{T_1\downarrow \quad T_2\downarrow}{T_1 \parallel T_2\downarrow} \quad \frac{\mathsf{sub}(E, E(X))\downarrow}{\langle X \,|\, E\rangle\downarrow}$$

(b) Termination

Fig. 3: Operational semantics of terms

$T_1 \parallel T_2$ is reduced by reducing $T_1$ and $T_2$ interleaved; and term $\langle X \,|\, E\rangle$ is reduced by reducing the version of $E(X)$ where recursion variables have been substituted.

A term is $\boxed{1}$-*free* if it has no occurrences of $\boxed{1}$. A term is *closed* if it has no occurrences of free recursion variables. A term $T$ is *deterministic* if (1) for every action $\alpha$, there exists at most one term $T'$ such that $T$ can reduce to $T'$ by performing $\alpha$, and (2) every term to which $T$ can reduce is deterministic as well. Henceforth, we consider only $\boxed{1}$-free, closed, and deterministic terms.

We note that $\langle \mathbb{A}, +, \cdot, \parallel\rangle$ is the signature of PA [3], while $\langle \boxed{1}, \mathbb{A}, +, \cdot, \parallel, \mathbb{X}, \langle - | - \rangle\rangle$ is a subsignature of TCP+REC [2]. As the operational semantics of terms in $\mathbb{T}\text{ERM}(\mathbb{A})$ coincides with the operational semantics of terms in (the corresponding subalgebra of) TCP+REC, our languages of global and local types inherit TCP+REC's sound and complete *axiomatisation*, used in our tool (Sect. 4.1).

#### 3.2 Global Types and Local Types

**Actions.** We instantiate $\mathbb{T}\text{ERM}(\mathbb{A})$ to obtain languages of global and local types by defining action sets for (global) communications and for (local) sends/receives.

Let $\mathbb{R} = \{\mathsf{a}, \mathsf{b}, ...\}$ denote the set of all *role names*, ranged over by $r$. Let $\mathbb{L}\text{AB} = \{\mathsf{Lock}, \mathsf{Get}, ...\}$ denote the set of all *labels*, ranged over by $\ell$. Let $\mathbb{T} = \{\mathsf{Nat}, \mathsf{Bool}, \ldots\}$ denote the set of all *payload types*, ranged over by $t$. Let $\mathbb{U} = \mathbb{L}\text{AB} \times \mathbb{T}$ denote the set of all *message types*, ranged over by $U$; we write $\ell(t)$ instead of $\langle \ell, t\rangle$. Finally, let $\mathbb{A}_g$ and $\mathbb{A}_l$ denote the sets of all *(global) communications* and *(local) sends/receives*, ranged over by $g$ and $l$, generated by:

$$g ::= r_1 \twoheadrightarrow r_2 : U \quad (\textbf{if: } r_1 \neq r_2)$$
$$l ::= r_1 r_2 ! U \mid r_1 r_2 ? U \mid \varepsilon^{r}_{r_1 r_2} \quad (\textbf{if: } r_1 \neq r_2 \textbf{ and } r_1 \neq r \neq r_2)$$

Global action $r_1 \twoheadrightarrow r_2 : U$ specifies the communication of a $U$-message from sender $r_1$ to receiver $r_2$; we note that communications are synchronous, as actions in the underlying algebra are indivisible [2,3], but asynchrony can be encoded (Exmp. 1, below). Local action $r_1 r_2 ! U$ specifies the send of a $U$-message through channel $r_1 r_2$ (from $r_1$ to $r_2$). Dually, local action $r_1 r_2 ? U$ specifies a receive. Local

$$\mathsf{split}(r, r_1 \twoheadrightarrow r_2 : U) = \begin{cases} (\boxed{1}, r_1 \twoheadrightarrow r_2 : U) & \textbf{if: } r \in \{r_1, r_2\} \\ (r_1 \twoheadrightarrow r_2 : U, \boxed{1}) & \textbf{otherwise} \end{cases}$$

$$\mathsf{split}(r, G_1 \cdot G_2) = \begin{cases} (G_1', G_1'' \cdot G_2) & \textbf{if: } \mathsf{split}(r, G_1) = (G_1', G_1'') \textbf{ and } G_1'' \neq \boxed{1} \\ (G_1 \cdot G_2', G_2'') & \textbf{if: } \mathsf{split}(r, G_1) = (G_1', G_1'') \textbf{ and } G_1'' = \boxed{1} \textbf{ and} \\ & \quad \mathsf{split}(r, G_2) = (G_2', G_2'') \textbf{ and } G_2'' \neq \boxed{1} \\ (G_1 \cdot G_2, \boxed{1}) & \textbf{otherwise} \end{cases}$$

$$\frac{}{G \rightsquigarrow G} \qquad \frac{M \rightsquigarrow G \quad \mathsf{split}(r_2, G) = (G', G'')}{r_1 \twoheadrightarrow r_2 : U \cdot M \rightsquigarrow r_1 \twoheadrightarrow r_1 r_2 : U \cdot \big[r_1 r_2 \twoheadrightarrow r_2 : U \parallel G'\big] \cdot G''} \text{ (asynchrony)}$$

$$\frac{}{\Sigma\, \emptyset \rightsquigarrow \boxed{1}} \qquad \frac{M_k \rightsquigarrow G_k \quad \Sigma\, \{M_i\}_{i \in I \setminus \{k\}} \rightsquigarrow G \quad k \in I}{\Sigma\, \{M_i\}_{i \in I} \rightsquigarrow G_k + G} \text{ (n-ary choice)}$$

$$\frac{}{\overline{\mu}(X, \ell_{\mathbf{c}}, \ell_{\mathbf{e}}, \emptyset) \rightsquigarrow \boxed{1}} \text{ (finite recursion: base)}$$

$$\frac{\begin{array}{c} M_i' = \overline{\mu}(X, \ell_{\mathbf{c}}, \ell_{\mathbf{e}}, \{\langle r_{1j}, r_{2j}, M_j \rangle\}_{j \in I \setminus \{i\}}) \textbf{ for all } i \in I \\ \Sigma\, \{\big[r_{1i} \twoheadrightarrow r_{2i} : \ell_{\mathbf{c}} \cdot M_i \cdot X\big] + \big[r_{1i} \twoheadrightarrow r_{2i} : \ell_{\mathbf{e}} \cdot M_i'\big]\}_{i \in I} \rightsquigarrow G \end{array}}{\overline{\mu}(X, \ell_{\mathbf{c}}, \ell_{\mathbf{e}}, \{\langle r_{1i}, r_{2i}, G_i \rangle\}_{i \in I}) \rightsquigarrow \mu X.\, G} \text{ (finite recursion: step)}$$

$$\frac{\Sigma\, \{M[r_i/r]\}_{i \in I} \rightsquigarrow G}{\exists r {\in} \{r_i\}_{i \in I}.\, M \rightsquigarrow G} \text{ (existential role quantification)}$$

Fig. 4: Macros action $\varepsilon^{r}_{r_1 r_2}$ specifies the *idling* of role $r$ during a communication between roles $r_1$ and $r_2$. The inclusion of such annotated idling actions in local types is novel; we shortly elaborate on its purpose.

We can now define $\mathbb{G}\text{LOB} = \mathbb{T}\text{ERM}(\mathbb{A}_g)$ and $\mathbb{L}\text{OC} = \mathbb{T}\text{ERM}(\mathbb{A}_l)$ as the sets of all global and local types, ranged over by $G$ and $L$.

**Macros.** As a testimony to the unique expressive power of our language of global types, we extend it with a number of *macros* that can be expanded to "normal" global types in $\mathbb{G}\text{LOB}$. A macro $M$ is generated by the following grammar:

$$\begin{array}{l} M ::= G \in \mathbb{G}\text{LOB} \;\mid\; r_1 \twoheadrightarrow r_2 \cdot M \;\mid\; \Sigma\{M_i\}_{i \in I} \;\mid \\ \qquad\quad \overline{\mu}(X, \ell_{\mathbf{c}}, \ell_{\mathbf{e}}, \{\langle r_{1i}, r_{2i}, M_i \rangle\}_{i \in I}) \;\mid\; \exists r {\in} \{r_i\}_{i \in I}.\, M \end{array}$$

Degenerate "macro" $G$ is a normal global type; it is part of the grammar to nest global types inside macros. Macro $r_1 \twoheadrightarrow r_2 \cdot M$ specifies an asynchronous communication from sender $r_1$ to receiver $r_2$. Macro $\Sigma\{M_i\}_{i \in I}$ specifies an n-ary choice among $|I|$ alternatives. Macro $\overline{\mu}(X, \ell_{\mathbf{c}}, \ell_{\mathbf{e}}, \{\langle r_{1i}, r_{2i}, M_i \rangle\}_{i \in I})$ specifies finite recursion: at the start of each unfolding of recursion variable $X$, for some $i \in I$, either an $\ell_{\mathbf{c}}$-message is communicated from sender $r_{1i}$ to receiver $r_{2i}$ (in which case they ***c**ontinue* their participation in the recursion), or an $\ell_{\mathbf{e}}$-message is communicated (in which case they ***e**xit*). Macro $\exists r {\in} \{r_i\}_{i \in I}.\, M$ specifies existential role quantification. Macros can be nested. Slightly abusing notation, we allow macros to occur and be expanded freely in "normal" global types.

Fig. 4 defines the macro expansion rules. We note that the left-hand side of $\rightsquigarrow$ is a macro, while the right-hand side is a normal global type. We demonstrated existential role quantification in Sect. 2; below, we give two more examples to illustrate our encoding of asynchronous communication and finite recursion.

*Example 1 (Asynchrony).* Although communications are synchronous, we can encode asynchrony by representing *buffered channels* (unordered, as in asynchronous $\pi$-calculus [32]) explicitly as roles that participate in a protocol. To this end, assume for all $r_1, r_2 \in \mathbb{R}$, there exists a role $r_1 r_2 \in \mathbb{R}$ as well (to represent the buffer from $r_1$ to $r_2$); alternatively $r_1 r_2$ could be any fresh name.

The following global types (message types omitted) specify paradigmatic cases for protocols with asynchronous communications:

$$
\begin{aligned}
M_1 &= \mathsf{a} \twoheadrightarrow \mathsf{b} \cdot \boxed{1} && \rightsquigarrow G_1 = \mathsf{a} \rightarrowtriangle \mathsf{ab} \cdot \mathsf{ab} \rightarrowtriangle \mathsf{b} \\
M_2 &= \mathsf{a} \twoheadrightarrow \mathsf{b} \cdot \mathsf{a} \twoheadrightarrow \mathsf{b} \cdot \boxed{1} && \rightsquigarrow G_2 = \mathsf{a} \rightarrowtriangle \mathsf{ab} \cdot \big[\mathsf{ab} \rightarrowtriangle \mathsf{b} \parallel \mathsf{a} \rightarrowtriangle \mathsf{ab}\big] \cdot \mathsf{ab} \rightarrowtriangle \mathsf{b} \\
M_3 &= \mathsf{a} \twoheadrightarrow \mathsf{b} \cdot \mathsf{b} \twoheadrightarrow \mathsf{a} \cdot \boxed{1} && \rightsquigarrow G_3 = \mathsf{a} \rightarrowtriangle \mathsf{ab} \cdot \mathsf{ab} \rightarrowtriangle \mathsf{b} \cdot \mathsf{b} \rightarrowtriangle \mathsf{ba} \cdot \mathsf{ba} \rightarrowtriangle \mathsf{a} \\
M_4 &= \mathsf{a} \twoheadrightarrow \mathsf{b} \cdot \mathsf{a} \rightarrowtriangle \mathsf{b} && \rightsquigarrow G_4 = \mathsf{a} \rightarrowtriangle \mathsf{ab} \cdot \mathsf{ab} \rightarrowtriangle \mathsf{b} \cdot \mathsf{a} \rightarrowtriangle \mathsf{b}
\end{aligned}
$$

(For brevity, we omit $\boxed{1}$ from the resulting global types; this can be incorporated in the macro expansion rules, at the expense of a more complex formulation.)

Global type $G_1$ specifies an asynchronous communication from Alice to Bob. Global type $G_2$ specifies two asynchronous communications from Alice to Bob; Alice can do the second send already *before* Bob has done the first receive. Global type $G_3$ specifies an asynchronous communication from Alice to Bob, followed by one from Bob to Alice; in contrast to $G_2$, Bob can send only *after* he has received (i.e., this encoding of asynchrony preserves causality of messages sent and received by the same role). Global type $G_4$ specifies an asynchronous communication from Alice to Bob, followed by a *synchronous* communication from Bob to Alice; it highlights that, unlike existing languages of global types, ours supports mixing synchrony and asynchrony in a single global type. □

*Example 2 (Finite recursion).* The Key-Value Store protocol in Sect. 2 does not terminate: in its global type, the inner recursions ($Y$ and $Z$) can be exited, but the outer recursion ($X$) cannot. A version of this protocol that terminates once each of the Clients has indicated it has finished using the store (e.g., by sending an $\mathsf{Exit}$-message) can also be specified.

We illustrate the key idea in a simplified example:

$$
G_1 = \mu X.\big[\big[\mathsf{a} \rightarrowtriangle \mathsf{c} : \mathsf{Con} \cdot X\big] + \mathsf{a} \rightarrowtriangle \mathsf{c} : \mathsf{Exit}\big] \quad G_2 = \mu X.\big[\big[\mathsf{b} \rightarrowtriangle \mathsf{c} : \mathsf{Con} \cdot X\big] + \mathsf{b} \rightarrowtriangle \mathsf{c} : \mathsf{Exit}\big]
$$
$$
G = \mu X.\big[\big[\mathsf{a} \rightarrowtriangle \mathsf{c} : \mathsf{Con} \cdot X\big] + \big[\mathsf{a} \rightarrowtriangle \mathsf{c} : \mathsf{Exit} \cdot G_2\big]\big] + \big[\mathsf{b} \rightarrowtriangle \mathsf{c} : \mathsf{Con} \cdot X\big] + \big[\mathsf{b} \rightarrowtriangle \mathsf{c} : \mathsf{Exit} \cdot G_1\big]
$$

Global type $G_1$ specifies the communication of either a $\mathsf{Con}$-message (to continue the recursion) or an $\mathsf{Exit}$-message (to break it) from Alice to Carol. Global type $G_2$ is similar. Global type $G$ specifies the communication of a $\mathsf{Con}$-message from

$$\frac{\mathcal{L}(r)\downarrow \quad \textbf{for all } r \in \operatorname{dom}\mathcal{L}}{\mathcal{L}\downarrow}$$

(a) Termination

$$\frac{\mathcal{L}(r_1) \xrightarrow{r_1 r_2 \,!\, U} L'_{r_1} \quad \mathcal{L}(r_2) \xrightarrow{r_1 r_2 \,?\, U} L'_{r_2}}{\mathcal{L} \xrightarrow{r_1 \twoheadrightarrow r_2 : U} \mathcal{L}[r_1 \mapsto L'_{r_1}, r_2 \mapsto L'_{r_2}]} \qquad \frac{\mathcal{L}(r) \xrightarrow{\varepsilon^r_{r_1 r_2}} L'_r}{\mathcal{L} \xrightarrow{\varepsilon^r_{r_1 r_2}} \mathcal{L}[r \mapsto L'_r]}$$

(b) Reduction

Fig. 5: Operational semantics of groups of local types

$$T \upharpoonright r = T \quad \textbf{if: } G \in \{\mathbf{1}\} \cup \mathbb{X}$$

$$(G_1 * G_2) \upharpoonright r = (G_1 \upharpoonright r) * (G_2 \upharpoonright r) \quad \textbf{if: } * \in \{+, \cdot, \|\}$$

$$r_1 \twoheadrightarrow r_2 : U \upharpoonright r = \begin{cases} r_1 r_2 ! U & \textbf{if: } r_1 = r \neq r_2 \\ r_1 r_2 ? U & \textbf{if: } r_1 \neq r = r_2 \\ \varepsilon^r_{r_1 r_2} & \textbf{if: } r_1 \neq r \neq r_2 \end{cases}$$

$$\langle X \,|\, E \rangle \upharpoonright r = \langle X \,|\, E \upharpoonright r \rangle \qquad E \upharpoonright r = \{X \mapsto E(X) \upharpoonright r \mid X \in \operatorname{dom} E\}$$

$$G \upharpoonright\upharpoonright R = \{r \mapsto G \upharpoonright r \mid r \in R\} \quad \textbf{if: } \mathsf{r}(G) \subseteq R \neq \emptyset$$

Fig. 6: Projection either Alice or Bob to Carol, or an Exit-message. In the latter case, Carol stops communicating with a role, while she proceeds communicating with the other role. Thus, the communications between Alice and Carol, and between Bob and Carol, are decoupled (i.e., decisions to continue or break recursions are made per role). Macro $\overline{\mu}$ generalizes this pattern to arbitrary recursion bodies. □

**Groups.** Finally, let $\mathbb{R} \rightharpoonup \mathbb{L}\text{OC}$ denote the set of all *groups* of local types (i.e., every group is a partial function from role names to local types), ranged over by $\mathcal{L}$. The idea is that while a global type specifies a protocol among $n$ roles from one global perspective, a group of local types specifies a protocol from the $n$ local perspectives. Fig. 5 defines the operational semantics of groups, built on top of the operational semantics of local types; we use the $f[x \mapsto y]$ notation to update function $f$ with entry $x \mapsto y$. In words, group $\mathcal{L}$ is reduced either by synchronously reducing the local types of a sender $r_1$ and a receiver $r_2$ (yielding a communication from $r_1$ to $r_2$), or by reducing the local type of an idling role.

#### 3.3 End-Point Projection: from Global Types to Local Types

A key part of MPST (Fig. 1) is a projection operator that consumes a global type $G$ as input and produces a group of local types $\mathcal{L}$ as output; it is *correct* if, under certain well-formedness conditions, $G$ and $\mathcal{L}$ are operationally equivalent.

Let $\mathsf{r}(G)$ denote the set of all role names that occur in $G$. Fig. 6 defines our projection operator. In words, the projection of a communication $r_1 \twoheadrightarrow r_2 : U$ onto a role $r$ is a send $r_1 r_2 ! U$ if the role is sender in the communication, a receive $r_1 r_2 ? U$ if it is receiver, or an idling action $\varepsilon^r_{r_1 r_2}$ if it is not involved; the projections of all other forms of global types onto $r$ are homomorphic; the projection of a global type onto a set of roles $R$ is the corresponding group of

$$\frac{T\downarrow}{T\Downarrow} \qquad \frac{T \xrightarrow{\tau} T' \Downarrow}{T\Downarrow} \qquad\qquad \frac{T \xrightarrow{\alpha} T'}{T \xRightarrow{\alpha} T'} \qquad \frac{T \xrightarrow{\tau} T' \xRightarrow{\alpha} T''}{T \xRightarrow{\alpha} T''} \qquad \frac{T \xRightarrow{\alpha} T' \xrightarrow{\tau} T''}{T \xRightarrow{\alpha} T''} \qquad \frac{T \xRightarrow{\sigma} T'}{T \xRightarrow{\tau} T'}$$

(a) Termination (b) Reduction

Fig. 7: Weak operational semantics; $T, T', T'' \in \mathbb{G}\text{LOB} \cup \mathbb{L}\text{OC} \cup (\mathbb{R} \rightharpoonup \mathbb{L}\text{OC})$

projections, where the side condition implies that the group is nonempty and contains a local type for at least every role name that occurs in $G$. Thus, a group of projections of $G$ is a *partial* function relative to the set of all roles $\mathbb{R}$, but it is *total* relative to the set of roles $\mathsf{r}(G) \subseteq \mathbb{R}$ that occur in $G$. (We note that we also continue to assume global types are $\mathbf{1}$-free, closed, and deterministic.)

Our projection operator is similar to existing projection operators in the MPST literature [34], but it also differs on a fundamental account: it produces local types with annotated idling actions. These idling actions will be instrumental in the definition of our well-formedness conditions. We note that no idling actions occur in the local types for the Key-Value Store protocol in Sect. 2. This is because *after* the idling actions have been used to establish well-formedness, they are of no more use and can be eliminated to simplify the local types.

The following lemmas state key properties about termination and reduction behaviour of global types and their projections: Lem. 1 states projection is sound and complete for termination; Lem. 2 states the same for reduction.

**Lemma 1.** $\big[G\downarrow$ **implies** $(G \upharpoonright r)\downarrow\big]$ **and** $\big[(G \upharpoonright r)\downarrow$ **implies** $G\downarrow\big]$

*Proof.* By induction on $G$. □

**Lemma 2.** $\big[G \xrightarrow{g} G'$ **implies** $(G \upharpoonright r) \xrightarrow{g\upharpoonright r} (G' \upharpoonright r)\big]$
**and** $\big[(G \upharpoonright r) \xrightarrow{g\upharpoonright r} L'$ **implies** $\big[\big[G \xrightarrow{g} G'$ **and** $L = G' \upharpoonright r\big]$ **for some** $G'\big]\big]$

*Proof.* Both conjuncts are proven by induction on the structure of $G$, also using Lem. 1 (needed because termination plays a role in reduction of $\cdot$). □

#### 3.4 Weak Bisimilarity of Global Types, Local Types, and Groups

The idling actions introduced in local types by our projection operator are *internal*, because they never compose into communications that emerge between local types in groups. Therefore, the operational equivalence relation under which we prove the correctness of projection should be insensitive to idling actions.

First, let $\mathbb{A}_\tau = \{\varepsilon^{r}_{r_1 r_2} \mid r_1 \neq r_2$ **and** $r_1 \neq r \neq r_2\}$ denote the set of all *internal actions*, ranged over by $\tau, \sigma$. Second, Fig. 7 defines an extension of our operational semantics (Fig. 3) with relations that assert *weak termination* and *weak reduction* (i.e., versions of termination and reduction that are insensitive to internal actions). Third, Fig. 8 defines weak bisimilarity ($\approx$), in terms of weak similarity ($\preceq$), in terms of weak termination and weak reduction; it coincides with the definition found in the literature (e.g., [2]), with the administrative

$$\frac{T_1\downarrow \textbf{ implies } T_2 \Downarrow \qquad \begin{bmatrix} [[T_1' \preceq T_2' \textbf{ and } T_2 \overset{\alpha}{\Longrightarrow} T_2'] \textbf{ for some } T_2'] \\ \textbf{or } [T_1' \preceq T_2 \textbf{ and } \alpha \in \mathbb{A}_\tau] \end{bmatrix} \textbf{ for all } T_1 \overset{\alpha}{\longrightarrow} T_1'}{T_1 \preceq T_2} \qquad \frac{\mathcal{R}, \mathcal{R}^{-1} \subseteq \preceq \quad T_1 \mathrel{\mathcal{R}} T_2}{T_1 \approx T_2}$$

Fig. 8: Weak operational equivalence; $T_1, T_1', T_2, T_2' \in \mathbb{G}\text{LOB} \cup \mathbb{L}\text{OC} \cup (\mathbb{R} \rightharpoonup \mathbb{L}\text{OC})$

exception that we need the fourth rule in Fig. 7b to account for the fact we have multiple different internal actions. We use a double horizontal line in the formulation of rules to indicate they should be applied coinductively.

The notion of weak reduction allows us to generalize the soundness and completeness of projection from roles (Lem. 2) to groups of roles: Lem. 3 states (1) if $G$ can $g$-reduce to $G'$ and the projection of $G'$ is defined, then the group of projections of $G$ can reduce to the group of projections of $G'$, either directly or with a trailing weak $\tau$-reduction; (2) conversely, if the group of projections of $G$ can $g$-reduce to $\mathcal{L}'$, then $G$ can $g$-reduce to $G'$ and either $\mathcal{L}'$ equals the group of projections of $G'$, or it can get there with a weak reduction.

**Lemma 3.** $\left[\begin{bmatrix} G \overset{g}{\longrightarrow} G' \textbf{ and} \\ G \upharpoonright\!\upharpoonright R \textit{ is defined} \end{bmatrix} \textbf{ implies } \begin{bmatrix} \begin{bmatrix} (G \upharpoonright\!\upharpoonright R) \overset{g}{\longrightarrow} (G' \upharpoonright\!\upharpoonright R) \textbf{ or} \\ (G \upharpoonright\!\upharpoonright R) \overset{g}{\longrightarrow} \mathcal{L}' \overset{\tau}{\Longrightarrow} (G' \upharpoonright\!\upharpoonright R) \end{bmatrix} \\ \textbf{for some } \mathcal{L}', \tau \end{bmatrix}\right]$

**and** $\left[(G \upharpoonright\!\upharpoonright R) \overset{g}{\longrightarrow} \mathcal{L}' \textbf{ implies } \begin{bmatrix} \left[G \overset{g}{\longrightarrow} G' \textbf{ and } \begin{bmatrix} \mathcal{L}' = G' \upharpoonright\!\upharpoonright R \textbf{ or} \\ \mathcal{L}' \overset{\tau}{\Longrightarrow} (G' \upharpoonright\!\upharpoonright R) \end{bmatrix}\right] \\ \textbf{for some } G', \tau \end{bmatrix}\right]$

*Proof.* Both conjuncts are proven by induction on $R$, also using Lem. 2. □

#### 3.5 Well-formedness of Global Types

In general, projection does not preserve weak operational semantics.

*Example 3 (Bad protocols).* The following global types (message types omitted) specify "bad" protocols that do not permit "good" concurrent implementations:

$$G_1 = \mathsf{a} \twoheadrightarrow \mathsf{b} + \mathsf{a} \twoheadrightarrow \mathsf{c} \qquad\qquad G_2 = \mathsf{a} \twoheadrightarrow \mathsf{b} \cdot \mathsf{c} \twoheadrightarrow \mathsf{d}$$

$$\underbrace{\mathsf{ab}! + \mathsf{ac}!}_{G_1 \upharpoonright \mathsf{a}} \quad \underbrace{\mathsf{ab}? + \varepsilon^{\mathsf{b}}_{\mathsf{ac}}}_{G_1 \upharpoonright \mathsf{b}} \quad \underbrace{\varepsilon^{\mathsf{c}}_{\mathsf{ab}} + \mathsf{ac}?}_{G_1 \upharpoonright \mathsf{c}} \qquad \underbrace{\mathsf{ab}! \cdot \varepsilon^{\mathsf{a}}_{\mathsf{cd}}}_{G_2 \upharpoonright \mathsf{a}} \quad \underbrace{\mathsf{ab}? \cdot \varepsilon^{\mathsf{a}}_{\mathsf{cd}}}_{G_2 \upharpoonright \mathsf{b}} \quad \underbrace{\varepsilon^{\mathsf{c}}_{\mathsf{ab}} \cdot \mathsf{cd}!}_{G_2 \upharpoonright \mathsf{c}} \quad \underbrace{\varepsilon^{\mathsf{d}}_{\mathsf{ab}} \cdot \mathsf{cd}?}_{G_2 \upharpoonright \mathsf{d}}$$

Global type $G_1$ specifies a communication from Alice to either Bob or Carol, chosen by Alice. This is a bad protocol, because if Alice chooses Bob, there is no way for Carol to know (and vice versa): Carol cannot locally distinguish between whether Alice has not made her choice yet, or whether Alice has chosen Bob. Formally, this is manifested in the fact that Carol's local type can *at any time*

choose to perform idling action $\varepsilon^{\mathsf{c}}_{\mathsf{ab}}$ (i.e., local type $G_1 \upharpoonright \mathsf{c}$ has two reductions, neither one of which has priority), thereby *assuming* that Alice has chosen Bob. However, Bob can symmetrically assume that Alice has chosen Carol. As a result, the group projection can reduce as follows: $G_1 \upharpoonright\!\upharpoonright \{\mathsf{a}, \mathsf{b}, \mathsf{c}\} \xrightarrow{\varepsilon^{\mathsf{c}}_{\mathsf{ab}}} \mathcal{L}_1 \xrightarrow{\varepsilon^{\mathsf{b}}_{\mathsf{ac}}} \mathcal{L}_2$. Now, $\mathcal{L}_2$ cannot reduce further, but Alice has not terminated yet. This sequence of reductions cannot be (weakly) simulated by $G_1$.

Global type $G_2$ specifies a communication from Alice to Bob, followed by a communication from Carol to Dave. This is a bad protocol, because there is no way for Carol and Dave to know when the communication from Alice to Bob has occurred. Formally, this is manifested in the fact that Carol's and Dave's local types can *at any time* choose to perform idling actions, thereby *assuming* that the communication from Alice to Bob has occurred. As a result, the group projection can reduce as follows: $G_2 \upharpoonright\!\upharpoonright \{\mathsf{a}, \mathsf{b}, \mathsf{c}, \mathsf{d}\} \xrightarrow{\varepsilon^{\mathsf{c}}_{\mathsf{ab}}} \mathcal{L}_1 \xrightarrow{\varepsilon^{\mathsf{d}}_{\mathsf{ab}}} \mathcal{L}_2 \xrightarrow{\mathsf{d} \twoheadrightarrow \mathsf{d}} \mathcal{L}_3 \xrightarrow{\mathsf{a} \twoheadrightarrow \mathsf{b}} \mathcal{L}_4$. This sequence cannot be (weakly) simulated by $G_2$. □

Next, we define two well-formedness conditions that invalidate the previous examples; in Sect. 3.6, we prove that if these conditions are satisfied by a global type $G$, it is indeed guaranteed that $G$ and $G \upharpoonright\!\upharpoonright R$ are operationally equivalent (i.e., weakly bisimilar). Instead of defining the conditions in terms of global types, we define them in terms of projections (i.e., local types). Informally:

**C** For every $r \in R$, for every choice that local type $G \upharpoonright r$ has between a weak reduction $\overset{l}{\Rightarrow}$ (where $l$ is a send, a receive, or an idling action) and a completely unobservable weak reduction $\overset{\tau}{\Longrightarrow}$, choosing to perform the former does not disable the latter, and vice versa. This can be thought of as a form of *commutativity* between $l$ and $\tau$.

**EC** For every $r \in R$, one of the following is true:

1. For every every weak reduction $\overset{l}{\Rightarrow}$ that local type $G \upharpoonright r$ can perform (where $l$ is a send or a receive, but not an idling action), it can perform a reduction $\xrightarrow{l}$. That is, if $G \upharpoonright r$ can perform $l$ in the future after idling actions, it can do $l$ already *eagerly* in the present.
2. Local type $G \upharpoonright r$ is the start of a causal *chain*: a sequence of $\tau$-reductions, followed by a non-$\tau$-reduction, that are "causally related" to each other. An $\varepsilon^{r}_{r_1 r_2}$-reduction is causally related to a $\varepsilon^{r}_{r_3 r_4}$-reduction iff $\{r_1, r_2\} \cap \{r_3, r_4\} \neq \emptyset$. Globally speaking, this means communication between $r_3$ and $r_4$ must be preceded by communication between $r_1$ and $r_2$.

These conditions must hold coinductively for all local types that $G \upharpoonright r$ can reduce to. Essentially, these conditions state that by performing idling actions, a local type can neither *decrease* its possible behaviour (C), nor *increase* it (EC-1), unless it is guaranteed the added behaviour cannot be exercised yet, because it is causally related to other communications that need to happen first (EC-2).

*Example 4 (Bad protocols, continued).* Global type $G_1$ (Exmp. 3) is ill-formed: its projections onto $\mathsf{b}$ and $\mathsf{c}$ violate condition C. Global type $G_2$ (Exmp. 3) is also ill-formed: its projections onto $\mathsf{c}$ and $\mathsf{d}$ violate condition EC. □

$$\frac{\begin{array}{c}\left[\begin{array}{l}\left[\begin{array}{l}[\Lambda_1'' \approx \Lambda_2'' \textbf{ and } \Lambda_1' \overset{\alpha_2}{\Longrightarrow} \Lambda_1'' \textbf{ and } \Lambda_2' \overset{\alpha_1}{\Longrightarrow} \Lambda_2''] \textbf{ or}\\ [\Lambda_1'' \approx \Lambda_2' \textbf{ and } \Lambda_1' \overset{\alpha_2}{\Longrightarrow} \Lambda_1'' \textbf{ and } \alpha_1 \in \mathbb{A}_\tau] \textbf{ or}\\ [\Lambda_1' \approx \Lambda_2'' \textbf{ and } \Lambda_2' \overset{\alpha_1}{\Longrightarrow} \Lambda_2'' \textbf{ and } \alpha_2 \in \mathbb{A}_\tau] \textbf{ or}\\ [\Lambda_1' \approx \Lambda_2' \textbf{ and } \alpha_1, \alpha_2 \in \mathbb{A}_\tau]\end{array}\right]\\ \textbf{for some } \Lambda_1'', \Lambda_2''\end{array}\right]\\ \textbf{for all } [\Lambda \overset{\alpha_1}{\Longrightarrow} \Lambda_1' \textbf{ and } \Lambda \overset{\alpha_2}{\Longrightarrow} \Lambda_2']\end{array}}{\mathsf{C}^{\alpha_1}_{\alpha_2}(\Lambda)}
\qquad
\frac{\begin{array}{cc}\mathsf{C}^{\alpha}_{\tau}(\Lambda) & \mathsf{C}(\Lambda')\\ \textbf{for all } \alpha, \tau & \textbf{for all } \Lambda \overset{\alpha}{\longrightarrow} \Lambda'\end{array}}{\mathsf{C}(\Lambda)}$$

$$\frac{\begin{array}{c}\left[\begin{array}{l}\left[\begin{array}{l}[\Lambda'' \approx \Lambda^{**} \textbf{ and } \Lambda \overset{\alpha_2}{\longrightarrow} \Lambda^{*} \overset{\alpha_1}{\Longrightarrow} \Lambda^{**}] \textbf{ or}\\ [\Lambda'' \approx \Lambda^{*} \textbf{ and } \Lambda \overset{\alpha_2}{\longrightarrow} \Lambda^{*} \textbf{ and } \alpha_1 \in \mathbb{A}_\tau] \textbf{ or}\\ \mathsf{Chain}\, \Lambda\end{array}\right]\\ \textbf{for some } \Lambda^{*}, \Lambda^{**}\end{array}\right]\\ \textbf{for all } \Lambda \overset{\alpha_1}{\Longrightarrow} \Lambda' \overset{\alpha_2}{\longrightarrow} \Lambda''\end{array}}{\mathsf{EC}^{\alpha_1}_{\alpha_2}(\Lambda)}
\qquad
\frac{\begin{array}{cc}\mathsf{EC}^{\tau}_{\alpha}(\Lambda) & \mathsf{EC}(\Lambda')\\ \textbf{for all } \alpha \notin \mathbb{A}_\tau, \tau & \textbf{for all } \Lambda \overset{\alpha}{\longrightarrow} \Lambda'\end{array}}{\mathsf{EC}(\Lambda)}$$

$$\frac{\begin{array}{cc}[L_1' = L_2' \textbf{ and } l_1 = l_2] & [\mathsf{r}(\tau) \cap \mathsf{r}(l) \neq \emptyset \textbf{ and } [\mathsf{Chain}\, L' \textbf{ or } l \notin \mathbb{A}_\tau]]\\ \textbf{for all } [L \overset{l_1}{\longrightarrow} L_1' \textbf{ and } L \overset{l_2}{\longrightarrow} L_2'] & \textbf{for all } L \overset{\tau}{\longrightarrow} L' \overset{l}{\longrightarrow} L''\end{array}}{\mathsf{Chain}\, L}$$

Fig. 9: Well-formedness conditions; $\Lambda, \Lambda', \Lambda'', \Lambda_1', \Lambda_1'', \Lambda_2', \Lambda_2'' \in \mathbb{L}\text{OC} \cup (\mathbb{R} \rightharpoonup \mathbb{L}\text{OC})$

Fig. 9 defines $\mathsf{C}$ and $\mathsf{EC}$ formally. We define $\mathsf{C}$ not only for local types, but also for groups of local types, as this simplifies some notation later on. We prove key properties of $\mathsf{C}$: Thm. 1 states commutativity of local sends/receives/idling ($l$) in local types gets lifted to commutativity of global communications/idling ($\alpha$) in groups of local types; Lem. 4 states weak bisimilarity preserves commutativity.

**Theorem 1.** $\left[\left[\left[\begin{array}{c}\mathsf{C}^{l}_{\tau}(\mathcal{L}(r))\\ \textbf{for all } l, \tau\end{array}\right] \textbf{ for all } r \in \operatorname{dom}\mathcal{L}\right] \textbf{ implies } \left[\begin{array}{c}\mathsf{C}^{\alpha}_{\tau}(\mathcal{L})\\ \textbf{for all } \alpha, \tau\end{array}\right]\right]$
$\textbf{and } \left[[\mathsf{C}(\mathcal{L}(r)) \textbf{ for all } r \in \operatorname{dom}\mathcal{L}] \textbf{ implies } \mathsf{C}(\mathcal{L})\right]$

*Proof.* The first conjunct is proven by induction on the rules of $\Rrightarrow$. The second is proven by coinduction on the rule of $\mathsf{C}$, also using the first conjunct. □

**Lemma 4.** $\left[[\mathsf{C}^{\alpha_1}_{\alpha_2}(\mathcal{L}_1) \textbf{ and } \mathcal{L}_1 \approx \mathcal{L}_2] \textbf{ implies } \mathsf{C}^{\alpha_1}_{\alpha_2}(\mathcal{L}_2)\right]$
$\textbf{and } \left[[\mathsf{C}(\mathcal{L}_1) \textbf{ and } \mathcal{L}_1 \approx \mathcal{L}_2] \textbf{ implies } \mathsf{C}(\mathcal{L}_2)\right]$

*Proof.* The first conjunct is proven by applying the definitions of $\mathsf{C}$ and $\approx$; the second is proven by coinduction on the rule of $\mathsf{C}$, also using the first conjunct. □

We also prove key properties of $\mathsf{Chain}$ and $\mathsf{EC}$, both of which work *specifically* for groups of projections: Lem. 5 states if the projections of $r_1$ and $r_2$ are both causal chains, they cannot weakly reduce to local types where they can perform reciprocal actions ($r_1$ the send; $r_2$ the receive); Thm. 2 states eagerness of local sends/receives (not idling) in projections gets lifted to eagerness of global communications in groups of projections (cf. Thm. 1).

**Lemma 5.** $$\begin{bmatrix}\mathsf{Chain}\,(G \upharpoonright\!\upharpoonright R)(r_1) \overset{\tau_1}{\Longrightarrow} \mathcal{L}'(r_1) \xrightarrow{r_1 r_2\,!\,U} \mathcal{L}''(r_1)\ \textbf{and}\\ \mathsf{Chain}\,(G \upharpoonright\!\upharpoonright R)(r_2) \overset{\tau_2}{\Longrightarrow} \mathcal{L}'(r_2) \xrightarrow{r_1 r_2\,?\,U} \mathcal{L}''(r_2)\end{bmatrix}\ \textbf{implies false}$$

*Proof.* By induction on the rules of $\Rightarrow$. □

**Theorem 2.** $$\left[\begin{bmatrix}\mathsf{EC}^{\tau}_{l}((G \upharpoonright\!\upharpoonright R)(r))\\ \textbf{for all}\ l \notin \mathbb{A}_{\tau}, \tau, r \in R\end{bmatrix}\ \textbf{implies}\ \begin{bmatrix}\mathsf{EC}^{\tau}_{\alpha}(G \upharpoonright\!\upharpoonright R)\\ \textbf{for all}\ \alpha, \tau\end{bmatrix}\right]$$
$$\textbf{and}\ \left[\left[\mathsf{EC}(\mathcal{L}(r))\ \textbf{for all}\ r \in \mathrm{dom}\,\mathcal{L}\right]\ \textbf{implies}\ \mathsf{EC}(\mathcal{L})\right]$$

*Proof.* The first conjunct is proven by using Lem. 5; the second is proven by coinduction on the rule of EC, also using the first conjunct. □

We note that, in contrast to Lem. 4 for C, we do not have a lemma that states weak bisimilarity preserves EC. Such a lemma would have been highly useful in our subsequent proofs, but it is unfortunately false, because weak bisimilarity does not preserve Chain. A simple counterexample, for local types, is this: $L_1 = r_1 r_2 ! U$ and $L_2 = \varepsilon^{r_3}_{r_4 r_5} \cdot r_1 r_2 ! U$, where $\{r_1, r_2\} \cap \{r_3, r_4, r_5\} = \emptyset$. While $L_1$ and $L_2$ are weakly bisimilar, $L_1$ is the start of a unary causal chain, but $L_2$ is not. The problem here is that Chain depends on the role names associated with idling actions, whereas weak bisimilarity abstracts those role names away.

We call a global type *well-formed* if each of its projections satisfies C and EC.

#### 3.6 Correctness of Projection under Well-Formedness

We now to prove our main result: if a global type is well-formed, it is weakly bisimilar to the group of its projections. We start by defining a relation $\bowtie$ to relate global types with groups of local types (denoted by $\mathcal{R}$ in Fig. 8):

$$\frac{\mathsf{C}(G \upharpoonright\!\upharpoonright R) \quad \mathsf{EC}(G \upharpoonright\!\upharpoonright R) \quad (G \upharpoonright\!\upharpoonright R) \overset{\star}{\Longrightarrow} \mathcal{L}' \overset{\star}{\Longleftarrow} \mathcal{L} \quad \mathsf{C}(\mathcal{L})}{G \bowtie \mathcal{L}}$$

Here, we write $\mathcal{L}_1 \overset{\star}{\Longrightarrow} \mathcal{L}_2$ as an abbreviation for:

$$\left[\mathcal{L}_1 \approx \mathcal{L}'_1 \overset{\tau}{\Longrightarrow} \mathcal{L}'_2 \approx \mathcal{L}_2\ \textbf{for some}\ \mathcal{L}'_1, \mathcal{L}'_2\right]\ \textbf{or}\ \mathcal{L}_1 \approx \mathcal{L}_2$$

In words, $\mathcal{L}_1 \overset{\star}{\Longrightarrow} \mathcal{L}_2$ means $\mathcal{L}_1$ has a *silent reduction* (only $\tau$-s) to a term that is weakly bisimilar to $\mathcal{L}_2$, or $\mathcal{L}_1$ is already weakly bisimilar to $\mathcal{L}_2$ (without any reductions). Essentially, if $\mathsf{C}(G \upharpoonright\!\upharpoonright R)$ and $\mathsf{EC}(G \upharpoonright\!\upharpoonright R)$, then $\bowtie$ relates $G$ to a set of groups $S = \{\mathcal{L} \mid G \bowtie \mathcal{L}\}$ that can roughly be characterised as follows:

- (*base*) $G \upharpoonright\!\upharpoonright R$ is in $S$;
- (*successors*) any group to which $G \upharpoonright\!\upharpoonright R$ can silently reduce, is in $S$;
- (*predecessors*) any group that can silently reduce to $G \upharpoonright\!\upharpoonright R$, is in $S$;

– (*pseudo-predecessors*) any group that can silently reduce to a group to which $G \upharpoonright\!\!\upharpoonright R$ can silently reduce, is in $S$;
– (*closure*) $S$ is closed under weak bisimilarity.

The following technical lemma states if a well-formed group of projections $G \upharpoonright\!\!\upharpoonright R$ can weakly $g$-reduce to some group $\mathcal{L}'$, then the original global type $G$ can $g$-reduce to some $G'$, and $\mathcal{L}'$ and the group of projections of $G'$ either are weakly bisimilar, or they can weakly reduce to a weakly bisimilar group $\mathcal{L}''$.

**Lemma 6.** $\left[\left[\mathsf{C}(G \upharpoonright\!\!\upharpoonright R) \textbf{ and } \mathsf{EC}(G \upharpoonright\!\!\upharpoonright R) \textbf{ and } (G \upharpoonright\!\!\upharpoonright R) \overset{g}{\Longrightarrow} \mathcal{L}'\right]\right.$
$\left.\textbf{implies } \left[\left[G \xrightarrow{g} G' \textbf{ and } (G' \upharpoonright\!\!\upharpoonright R) \overset{\star}{\Longrightarrow} \mathcal{L}'' \overset{\star}{\Longleftarrow} \mathcal{L}'\right] \textbf{ for some } \mathcal{L}''\right]\right]$

*Proof.* By induction on the rules of $\Rightarrow$, also using Lem. 3. ☐

The following two lemmas state key properties of $\bowtie$: Lem. 7 states $\bowtie$ preserves termination (as weak termination); Lem. 8 states $\bowtie$ coinductively preserves reduction (as weak reduction). Together, these lemmas imply $\bowtie \subseteq \preceq$ and $\bowtie^{-1} \subseteq \preceq$, which in turn imply $\bowtie \subseteq \approx$.

**Lemma 7.** $\left[\left[G \bowtie \mathcal{L} \textbf{ and } G\downarrow\right] \textbf{ implies } \mathcal{L}\Downarrow\right]$
$\textbf{and } \left[\left[G \bowtie \mathcal{L} \textbf{ and } \mathcal{L}\downarrow\right] \textbf{ implies } G\Downarrow\right]$

*Proof.* The first conjunct is proven by induction on the rules of $\Rightarrow$, also using Lem. 1; the second is proven by contradiction (assume **not** $G\downarrow$; derive **false**; conclude $G\downarrow$; it implies $G\Downarrow$). ☐

**Lemma 8.** $\left[\left[G \bowtie \mathcal{L} \textbf{ and } G \xrightarrow{g} G'\right] \textbf{ implies } \left[\begin{matrix}\left[G' \bowtie \mathcal{L}' \textbf{ and } \mathcal{L} \overset{g}{\Longrightarrow} \mathcal{L}'\right] \\ \textbf{for some } \mathcal{L}'\end{matrix}\right]\right]$
$\textbf{and } \left[\left[G \bowtie \mathcal{L} \textbf{ and } \mathcal{L} \xrightarrow{g} \mathcal{L}'\right] \textbf{ implies } \left[\begin{matrix}\left[G' \bowtie \mathcal{L}' \textbf{ and } G \xrightarrow{g} G'\right] \\ \textbf{for some } G'\end{matrix}\right]\right]$
$\textbf{and } \left[\left[G \bowtie \mathcal{L} \textbf{ and } \mathcal{L} \xrightarrow{\tau} \mathcal{L}'\right] \textbf{ implies } G \bowtie \mathcal{L}'\right]$

*Proof.* The first and second conjunct are proven by induction on the rules of $\Rightarrow$, also using Lemmas 3–4; the third is proven by induction on the rules of $\Rightarrow$. ☐

**Theorem 3.** $\left[\mathsf{C}(G \upharpoonright\!\!\upharpoonright R) \textbf{ and } \mathsf{EC}(G \upharpoonright\!\!\upharpoonright R)\right] \textbf{ implies } G \approx (G \upharpoonright\!\!\upharpoonright R)$

*Proof.* By coinduction on the rule of $\preceq$ (Fig. 8), also using Lemmas 7-8. ☐

A group of local types $\mathcal{L}$ enjoys *deadlock-freedom* if it either has successfully terminated ($\mathcal{L}\downarrow$; Fig. 5a) or can make another reduction. A group of local types $\mathcal{L}$ enjoys *absence of protocol violations* relative to global type $G$ if, coinductively, every non-$\tau$ reduction of $\mathcal{L}$ can be simulated by $G$ (i.e., every communication in the group is "permitted" by $G$). The following corollary relates Thm. 3 of operational equivalence to these classical MPST properties:

**Corollary 1.** *If global type $G$ is well-formed, then the group of $G$'s projections enjoys deadlock-freedom and absence of protocol violations relative to $G$.*

The key insight to understand this, is that global types are *by definition* free of deadlocks (they either reduce to $\boxed{1}$, or they never terminate; Fig. 3), while weak bisimilarity preserves deadlock-freedom of global types in their projections (notably, weak bisimilarity is sensitive to termination, and a group of local types terminates only if *all* individual local types terminate; Fig. 5a). Weak bisimilarity also directly implies freedom of protocol violations.

#### 3.7 Decidability of Checking Well-Formedness

We note our proof of Thm. 3 is non-constructive, in the sense that $\bowtie$ is infinitely large (i.e., for each group of local types, there exist infinitely many weakly bisimilar groups). The following proposition states this is not a problem in practice.

**Proposition 1.** *Checking* $\mathsf{C}(\mathcal{L})$ *and* $\mathsf{EC}(\mathcal{L})$ *is decidable.*

The rationale behind this proposition is as follows. First, to check $\mathsf{C}(\mathcal{L})$ and $\mathsf{EC}(\mathcal{L})$, by Thm. 1 and Thm. 2, it suffices to check $\mathsf{C}(\mathcal{L}(r))$ and $\mathsf{EC}(\mathcal{L}(r))$ for each $r \in \operatorname{dom} \mathcal{L}$. For each such local type $\mathcal{L}(r)$, there are two possibilities.

If local type $\mathcal{L}(r)$ has finite control, its state space can be exhaustively explored in finite time, so checking $\mathsf{C}(\mathcal{L}(r))$ and $\mathsf{EC}(\mathcal{L}(r))$ is obviously decidable.

In contrast, if $\mathcal{L}(r)$ has non-finite control, we make two observations. The first observation is that the only possibly source of infinity is the occurrence of recursion variables under parallel composition. The second observation is that $\mathsf{C}$ and $\mathsf{EC}$ are true for $L_1 \parallel L_2$ if they are true for $L_1$ and $L_2$ separately; this is because $\mathsf{C}$ and $\mathsf{EC}$ essentially assert a "diamond structure" on the reductions of $L_1 \| L_2$, which is *precisely* the operational semantics of $\|$ (Fig. 3). Thus, we can check $\mathsf{C}(L_1 \| L_2)$ and $\mathsf{EC}(L_1 \| L_2)$ by checking $\mathsf{C}(L_1)$, $\mathsf{C}(L_2)$, $\mathsf{EC}(L_1)$, and $\mathsf{EC}(L_2)$, thereby "avoiding" the possible source of infinity.

We note that splitting the checks for parallel composition in this way not only ensures decidability; it also avoids exponential state explosion (in the number of nested $\|$-operators in a single local type) in local types with finite control.

#### 3.8 Discussion of Challenges

Our use of (weak) bisimilarity, plus the key insight to annotate silent actions with additional information to keep track of choices, made the problem of proving the correctness of projection (Thm. 3) feasible. The major technical challenges to achieve this were defining the right bisimulation relation (Sect. 3.5) and discovering corresponding well-formedness conditions (Sect. 3.6).

A naive weak bisimulation relation, $R_{\text{naive}}$, relates every global type only with its group of projections. $R_{\text{naive}}$ is sufficient to prove that every reduction of a global type can be weakly simulated with one non-silent reduction of the group (sender and receiver), followed by a number of silent reductions (idling

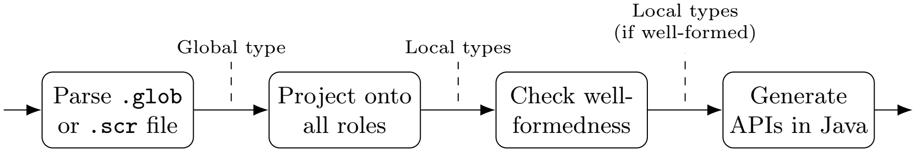

Fig. 10: Overview of `mpstpp`

processes). In contrast, $R_{\text{naive}}$ is insufficient to prove that every reduction of the group can be simulated by its global type, because of silent actions: if global type $G$ is related to group of projections $\mathcal{L}$ by $R_{\text{naive}}$, and a silent action subsequently reduces $\mathcal{L}$ to $\mathcal{L}'$, the simulation fails, as $R_{\text{naive}}$ does not relate $G$ to $\mathcal{L}'$.

To alleviate this issue, we defined the bisimulation relation in such a way that it relates every global type $G$ to a group of local types that are not necessarily *equal* to the projections of $G$, but every local type can be *behind* the corresponding projection (the local type can reach the projection with silent actions) or *ahead* (the projection can reach the local type with silent actions).

## 4 Practical Experience with the Theory

### 4.1 Implementation

**Tool.** We implemented a tool, `mpstpp`, based on the core theoretical contributions of this paper. Fig. 10 shows a high-level overview of the tool, including the main components (boxes) and data flows (arrows).

First, `mpstpp` parses an input .glob-file to a data structure for a global type $G$ (programmer-friendly Scribble-style syntax [35] is also supported as input). Then, it projects $G$ onto all roles that occur in $G$. Then, it checks each of the resulting local types for well-formedness, depending on settings, either sequentially or *in parallel*: a key advantage of the formulation of our well-formedness conditions is that they can be checked modularly for every role in isolation, enabling us to take advantage of modern multicore hardware. Finally, if the local types are well-formed, idling actions are eliminated and *typed communication APIs* are generated from the local types to enable MPST++-based programming in Java.

**Optimisations.** Parsing, computing projections, and generating APIs is relatively inexpensive; instead, the run times of our tool are dominated by checks for well-formedness. We therefore implemented several optimisations to make these checks more efficient. Before we present these optimisations, we first note that the complexity of checking well-formedness of a local type $L$ is polynomial in the number of *successors* that can be reached from $L$ (Fig. 9).

**(1)** Our first optimisation targets local types with parallel composition; local type $L_1 \parallel L_2$ is potentially a serious bottleneck, as its number of successors is *exponential* in the number of nested $\parallel$-operators. Therefore, even with finite state spaces, we check the well-formedness of $L_1 \parallel L_2$ by checking the well-formedness of $L_1$ and $L_2$, without explicitly considering the exponentially many successors of $L_1 \parallel L_2$, exploiting the same observation as with decidability (Sect. 3.7).

**(2)** Our second optimisation concerns computation of weak reductions. In particular, to check whether $\mathsf{C}$ and $\mathsf{EC}$ are true for a local type $L$, according to their definitions (Fig. 9), we need to iterate over each of their weak reductions. Especially if $L$ has many $\tau$-reductions (Fig. 7), computing the set of weak reductions can be expensive. To avoid this, `mpstpp` computes sound (but incomplete) *approximations* of $\mathsf{C}$ and $\mathsf{EC}$. We implemented two kinds of approximations: (a) checking versions of $\mathsf{C}$ and $\mathsf{EC}$ where every occurrence of $\Rightarrow$ in the definition is replaced with $\rightarrow$, and (b) checking $L \approx L'$ for every $\tau$-reduction from $L$ to $L'$. Approximation (a) is sound for both $\mathsf{C}$ and $\mathsf{EC}$ (rationale: if individual reductions can commute, sequences of reductions consisting of those individual reductions can commute as well), but approximation (b) is sound only for $\mathsf{C}$ (rationale: auxiliary relation $\mathsf{Chain}$ of $\mathsf{EC}$ is not preserved by weak bisimilarity). To ensure soundness, thus, `mpstpp` never uses approximation (b) for $\mathsf{EC}$.

**(3)** Our third optimisation targets the checks for weak bisimilarity that occur in several places in the definitions of $\mathsf{C}$ and $\mathsf{EC}$ (Fig. 9). Instead of computing the full reduction relations and run an algorithm to decide their weak bisimilarity (which would be computationally costly), we take advantage of the fact that our language of local types is based on existing algebras (Sect. 3.1) that have sound and complete axiomatisations. Specifically, to check whether two local types are weakly bisimilar, `mpstpp` applies the axioms as rewrite rules and compares the resulting normal forms for structural equality. To ensure rewriting is fast, we sacrificed completeness (i.e., we use rewriting only to eliminate as many silent actions as possible in a sound way, but for instance, our rewrite procedure cannot prove that $(L_1 \cdot \tau) + L_2$ and $L_2 + L_1$ are weakly bisimilar); however, for the ample examples we tried (including this paper's), this optimisation is highly effective.

Optimisations (2) and (3) are *conservative*: `mpstpp` may conclude $\mathsf{C}$ or $\mathsf{EC}$ is false, even though it is actually true. While this affects completeness, soundness is guaranteed: if `mpstpp` concludes a local type is well-formed, it really is.

#### 4.2 Evaluation of the Approach

**Setup.** In the previous section, we formulated and proved the theoretical *correctness* of our well-formedness conditions (Thm. 3). In this section, we demonstrate the practical *usefulness* through experimental evaluation in benchmarks. Specifically, we show that checking our well-formedness conditions is faster and more scalable than explicitly checking operational equivalence (which currently seems the only alternative to attain the same level of expressiveness as our work).

In our benchmarks, we compare three approaches to check operational equivalence between a global type and its group of projected local types:

– `mpstpp`-SEQ (baseline): In this approach, the `mpstpp` tool is used to check our well-formedness conditions (which imply operational equivalence; Thm. 3), without using any form of parallel processing.

– mpstpp-PAR: Like mpstpp-SEQ, except each projected local type is checked in a separate thread. The fact our well-formedness conditions can be easily parallelised in this way is an important practical advantage.
– EXPLICIT: In this approach, mpstpp is used only for parsing and projecting; after that, we use the state-of-the-art verification tool set mCRL2 [10,20,29] to explicitly check operational equivalence (details below).

We identified six example protocols (details below) that can naturally be scaled in the number of roles $N$ (e.g., the number of Clients in the Key-Value Store protocol). Using each of the three approaches, for each of the protocols, for each value of $N$ between the minimal number of roles $N_{\text{min}}$ (e.g, $N_{\text{min}}$=2 in the Key-Value Store protocol: the Server and one Client) and 16, we subsequently checked operational equivalence; varying $N$ in this way, yields insights not only in per-case performance, but also scalability. To get statistically reliable results [31], we repeated executions as many times as was necessary until the 95% confidence interval was within 5% of our reported means (i.e., there is a 95% probability that the true mean is within 5% of our reported means).

We ran our benchmarks on a machine with an Intel Xeon 6130 processor (16 cores; no hyper-threading), using Debian 9, Java 13, and mCRL2 201908.0.

**Translation to mCRL2.** In the EXPLICIT approach, we use mCRL2 [10,20,29] to explicitly check if global type $G$ and its group of projections $\mathcal{L}$ are operationally equivalent. Our choice for mCRL2 is motivated by the fact our languages of global and local types are based on the same process algebra as mCRL2's specification language, so their translation to mCRL2 specifications is direct and straightforward. Moreover, mCRL2 is mature (e.g., used in industry [5]), and it uses optimised, state-of-the-art algorithms to check behavioural equivalences (e.g., [28]), so we are comparing our tool with a serious competitor.

First, we translate global type $G$ to mCRL2 specification $[\![G]\!]$. Then, we use mCRL2 tools `mcrl22lps` and `lps2lts` to normalize $[\![G]\!]$ to a *linear process specification* (LPS) and generate a corresponding *labelled transition system* (LTS). Because of the directness of the translation, the transition labels in the resulting LTS are all global communication actions of the form $r_1 \twoheadrightarrow r_2 : U$.

Second, we translate group of projections $\mathcal{L}$, consisting of roles $r_1, ..., r_n$, to mCRL2 specification $[\![\mathcal{L}]\!]$. It looks as follows (in formal mCRL2 notation [29]):

$$\nabla_{\{r_i \twoheadrightarrow r_j : U \mid 1 \leq i,j \leq n, i \neq j, U \in \mathbb{U}\}}\big(\\ \Gamma_{\{(r_i r_j !U \sqcup r_i r_j ?U) \rightarrow (r_i \twoheadrightarrow r_j : U) \mid 1 \leq i,j \leq n, i \neq j, U \in \mathbb{U}\}}([\![\mathcal{L}(r_1)]\!] \parallel ... \parallel [\![\mathcal{L}(r_n)]\!])\big)$$

where each $[\![\mathcal{L}(r_i)]\!]$ is a direct translation of local type $\mathcal{L}(r_i)$ to an mCRL2 specification; $\parallel$ is a form of parallel composition that prescribes both interleaving and synchronisation of operand actions; $\sqcup$ is *synchronous composition* of actions; $\Gamma$ is the *communication operator* that replaces synchronised local send/receive actions $r_i r_j !U \sqcup r_i r_j ?U$ with global communication action $r_i \twoheadrightarrow r_j : U$; and $\nabla$ is the *allow operator* that allows only global communication actions to be executed (i.e., unsynchronized, individual send/receive actions cannot be executed).

When translating a local type $\mathcal{L}(r_i)$ to an mCRL2 specification $[\![\mathcal{L}(r_i)]\!]$, to make mCRL2's subsequent verification easier, we already eliminate as many idling actions $\varepsilon^{r}_{r_1 r_2}$ as possible (modulo branching bisimulation); those that remain are represented as a general $\tau$ action, because mCRL2 does not need the additional information provided by $\varepsilon^{r}_{r_1 r_2}$. Then, we use `mcrl22lps` and `lps2lts` to generate an LPS and LTS for $[\![\mathcal{L}]\!]$.

Third, we use mCRL2 tool `ltscompare` to check if the LTS for $[\![G]\!]$ is weakly bisimilar to the LTS for $[\![\mathcal{L}]\!]$. We note that normalisation to an LPS using `mcrl22lps` is a requirement to use `ltscompare`.

**Protocols.** We used the following protocols in our benchmarks:

***Key-Value Store*** (KVS): This protocol is the same protocol as the one presented in Sect. 2, except each inner parallel composition ($\|$) is replaced with sequential composition ($\cdot$). This is because `mcrl22lps` does not support normalisation of mCRL2 specifications where $\|$ occurs under recursion.

***Load Balancer*** (LB): This protocol consists of a *Master* and a number of *Workers*. Iteratively, first, a Request-message is communicated from the Master to one of the Workers; then, a Response-message is communicated from that Worker to the Master.

***Work Stealing*** (WS): This protocol consists of a *Master* and a number of *Workers*. Iteratively, a Job-message is communicated from the Master to one of the Workers. Meanwhile, Workers can try to "steal" jobs from each other: at any point, first, a Steal-message can be communicated from one Worker to another Worker; then, either a Job-message (if the former Worker has a job to spare) or a None-message (otherwise) is communicated from the latter Worker to the former Worker.

***Map/Reduce*** (MR): This protocol consists of a *Master* and a number of *Workers*. First, in no particular order, a Map-message is communicated from the Master to each Worker; then, in no particular order, a Reduce-message is communicated from each Worker to the Master.

***Peer-to-Peer*** (PtP): This protocol consists of a number of *Peers*. Unordered, a Msg-message is communicated from each Peer to each other Peer.

***Pub/Sub*** (PS): This protocol consists of a *Publisher* and a number of *Subscribers*. In no particular order, a Sub-message can be communicated once from each Subscriber to the Publisher to gain a subscription. Concurrently, a Pub-message can be communicated from the Publisher to each Subscriber with a subscription.

The table on the right summarises the features used in each of these protocols.

| | KVS | LB | WS | MR | PtP | PS |
|---|---|---|---|---|---|---|
| $+$ | ✓ | | ✓ | | | ✓ |
| $\exists$ | ✓ | ✓ | ✓ | | | ✓ |
| $\|$ | ✓ | | ✓ | ✓ | ✓ | |

For each $1 \le n \le 15$, we instantiated the Key-Value Store, Load Balancer, Work Stealing, and Map/Reduce protocols with 1 Server/Master + $n$ Clients/Workers. For each $2 \le n \le 16$, we instantiated the Peer-to-Peer protocol with $n$ Peers. For

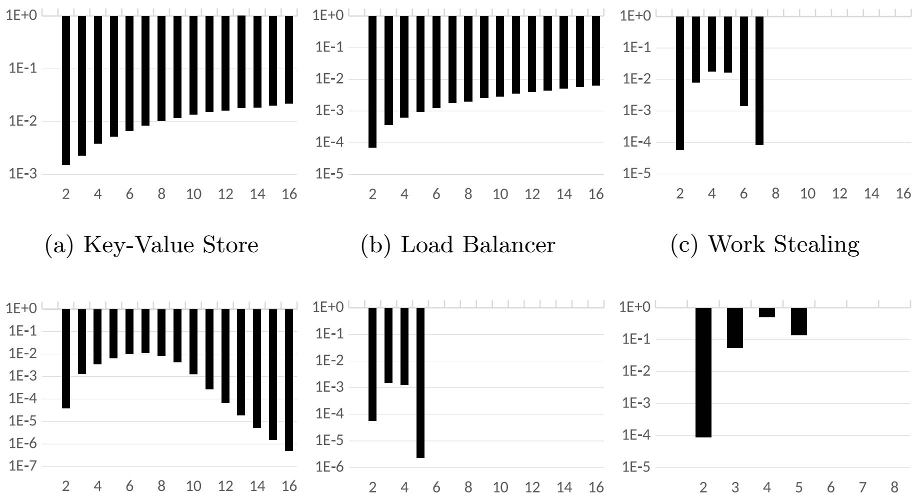

(a) Key-Value Store (b) Load Balancer (c) Work Stealing

(d) Map/Reduce (e) Peer-to-Peer (f) Pub/Sub

Fig. 11: Speedups ($y$-axis; $y$>1E+0 means faster, $y$<1E+0 means slower) of EXPLICIT relative to `mpstpp`-SEQ as the number of roles increases ($x$-axis)

each $2 \leq n \leq 7$, we instantiated the Pub/Sub protocol with 1 Publisher and $n$ Subscribers; we did not instantiate the Pub/Sub protocol with $n>7$ Subscribers, as the resulting global types are too large (their size grows exponentially in $n$).

**Benchmark results.** Figures 11–12 shows the results of our benchmarks. The $x$-axis indicates the number of roles; the $y$-axis indicates relative speed-ups. The baselines are at $y$=1E+0 and $y$=1: above it, a competing approach is *faster* than `mpstpp`-SEQ; below it, it is *slower*. We draw two conclusions.

**(1) For each protocol and number of roles, `mpstpp`-SEQ outperforms EXPLICIT.** In the cases of Key-Value Store and Load Balancer, EXPLICIT grows towards `mpstpp`-SEQ, but the growth levels off as the number of roles increases, while EXPLICIT is still about two order of magnitude slower than `mpstpp`-SEQ in the best of circumstances. In the cases of Work Stealing, Peer-to-Peer, and Pub/Sub, the LTSs generated from the translated mCRL2 specifications were too large to be compared (i.e., `ltscompare` produced an error) beyond 7, 5, and 5 roles; this was no issue for `mpstpp`-SEQ. In the case of Map/Reduce, the LTSs were small enough to compare using mCRL2's `ltscompare`, but after an initial upwards slope for $2 \leq N \leq 7$ roles, EXPLICIT starts to perform progressively worse.

**(2) Especially for larger numbers of roles, parallelisation can yield serious performance improvements.** In the cases of Key-Value Store and Load Balancer, `mpstpp`-PAR outperforms `mpstpp`-SEQ only with 14–16 roles; for smaller numbers of roles, parallel execution is slower. In the worst case (Load Balancer, 2 roles), the slowdown is roughly $\frac{10.9\mu s}{3.2\mu s} = 3.4$; we hypothesise that be-

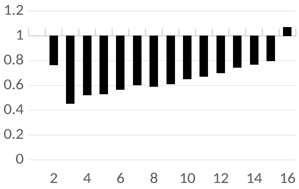

(a) Key-Value Store

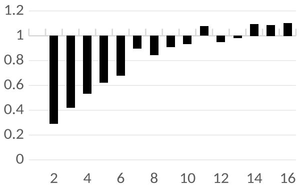

(b) Load Balancer

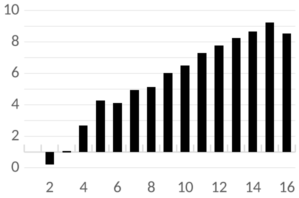

(c) Work Stealing

(d) Map/Reduce

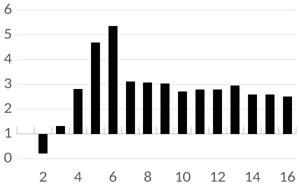

(e) Peer-to-Peer

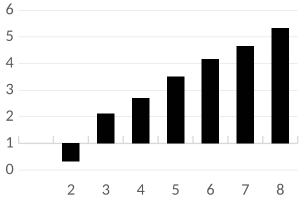

(f) Pub/Sub

Fig. 12: Speedups ($y$-axis; $y$>1 means faster, $y$<1 means slower) of `mpstpp`-PAR relative to `mpstpp`-SEQ as the number of roles increases ($x$-axis)

cause of the low absolute execution times, the cost of spawning and synchronising threads outweighs their benefit. However, the ascending gradient indicates that as the number of roles increases, relatively more of the total work can be parallelised, yielding progressive rewards. In the cases of Work Stealing, Map/Reduce, Peer-to-Peer, and Pub/Sub, similar trends can be observed, except $y$=1 is crossed sonner. The absolute execution times for these protocols and for small numbers of roles are higher than for Key-Value Store and Load Balancer.

### 5 Related Work

**Multiparty compatibility.** Closest to this paper is existing literature on multiparty compatibility [6,24,40,42]. The key idea, initially developed by Deniélou and Yoshida for the original MPST [23,24], is to represent (groups of) local types operationally as (*systems* of) *communicating finite state machines* (CFSM) [8]. A CFSM $M$ is a state machine where transitions are labelled with sends/receives; a system of CFSMs $S$ is a parallel composition where CFSMs communicate through asynchronous buffers. Multiparty compatibility, then, is a condition on the reachable states and transitions of a system $S = (M_1, ..., M_n)$: if it is satisfied by $S$, the system is guaranteed to be *safe* (no deadlocks; no unmatched sends/receives) and *live* ($S$ terminates, assuming at least one $M_i$ can terminate). Multiparty compatibility is a sufficient condition to guarantee safety and liveness, but not necessary: there exist safe/live systems that are not multiparty compatible. Therefore, several generalisations have been proposed to cover timed behaviour [6], undirected choice [40], and non-synchronisability [42].

The main similarities between our method in this paper and the multiparty compatibility approach are: (1) we also use an operational interpretation of local types; (2) we guarantee similar liveness/safety properties; (3) and we also neatly factor out the act of checking conformance of processes to local types (resp. CFSMs). In contrast, we support a wider range of behaviours. Moreover, from a practical/computational perspective, multiparty compatibility is a global condition that needs to be checked on the whole state space of a system (i.e., parallel composition of the CFSMs), prone to exponential blow-up; our well-formedness conditions, in contrast, are completely local and require only polynomial time to check. The reason we do not require CFSM-like machinery in this paper is that our operational correspondence (weak bisimilarity) is sensitive to termination: notably, in Fig. 5a, a group of local types terminates *iff* every individual local type terminates (for multiparty compatibility, proofs are done modulo trace equivalence [24], which cannot distinguish between successful/abnormal termination and is therefore in itself too weak to show deadlock-freedom).

**Expressiveness of MPST.** In the original MPST theory [33], and many of its descendants (e.g., [14,19,22,24,25,43]), the restrictions on choices are enforced through a combination of syntax and additional well-formedness conditions. Notably, in these works, communications in global types are specified as $r_1 \twoheadrightarrow r_2 : \{\ell_i \cdot G_i\}_{i \in I}$, so syntactically, it is impossible to specify choices among senders or receivers. There exist also papers where a seemingly more general binary +-like operator is introduced, particularly those that support choices among receivers [16,23,36,40], but the well-formedness conditions still basically restrict the use of + in these works to $r_1 \twoheadrightarrow r_2 : \{\ell_i \cdot G_i\}_{i \in I}$ or $r \twoheadrightarrow \{r_i : \ell_i \cdot G_i\}_{i \in I}$.

This is the first paper where well-formedness conditions do not force the use of + into one of those two restricted forms. Moreover, our well-formedness conditions are compatible with unbounded interleaving (recursion under parallel), beyond similar operators in previous work [16,22,23,43]. An alternative approach is to completely omit statically checked well-formedness conditions (and projection), and to only *dynamically* verify communication actions against global types through monitoring, as recently proposed [30]. The language of global types in that paper is more expressive than ours in this paper, but all verification happens at *run-time*, whereas we provide correctness guarantees already at *compile-time*.

**Session types and model checking.** Recently, there has been growing interest in using model checking to verify properties of (multiparty) session types, similar to our use of mCRL2 as an alternative to checking well-formedness (Sect. 4.2). Lange et al. [39] infer behavioural types from Go programs and use mCRL2 to verify the inferred types, to establish safety properties (combined with another tool, KITTeL [26], to establish liveness). Hu and Yoshida [36] use a custom model checker to verify safety and progress properties of local types (represented as CFSMs) as part of API generation in the Scribble toolchain for MPST [35].

Closest to our use of mCRL2 is the work of Scalas et al. [52,53], where mCRL2 is used to verify properties of local types (e.g., deadlock-freedom), while a form of dependent type-checking is used to verify conformance of processes against those types (i.e., actors in Scala); no global types and projection are used, though (programmers write local types manually). The idea is that properties model-checked on the types carry over to the processes. Similarly, Scalas and Yoshida [51] use mCRL2 to model-check session environments, as a more expressive alternative to the classical consistency condition needed to prove subject reduction. Note that [51, Theorem 5.15] shows that, in the case that a set of processes is typable by a single multiparty session (i.e. a single global type), type-level properties including safety, deadlock-freedom and liveness guarantee the same properties for multiparty session $\pi$-processes. Hence our type-level analysis is directly usable to provide decidable procedures to verify session $\pi$-calculi with extended expressiveness [51, Theorem 7.2].

### 6 Conclusion

A key open problems with multiparty session types (MPST) concerns expressiveness: none of the previous languages of global and local types supports arbitrary choice (e.g., choices between different senders), existential quantification over roles, and unbounded interleaving of subprotocols (in the same session). In this paper, we presented the first theory that supports these features. Our main theoretical result is operational equivalence under weak bisimilarity: this guarantees classical MPST properties for groups of local types projected from a global type, namely freedom of deadlocks and absence of protocol violations. Our main practical result is that our well-formedness conditions, which guarantee operational equivalence, can be checked orders of magnitude faster than directly checking weak bisimilarity, which is demonstrated by our benchmark results.

We identify several interesting avenues for future work. First, it is useful to extend our theory with *parametrisation* along the lines of Castro et al. [18] (which currently works only for restrictive choices); their proof technique for correctness seems to offer substantial synergy with our bisimilarity-based approach in this paper. Second, we aim to investigate extensions of our theory with subtyping (e.g., in terms of weak similarity). Notably, while asynchronous communication can be encoded in our current theory, asynchronous subtyping is known to be undecidable [9,41], so the connection between the two is interesting to explore.

*Acknowledgments.* Funded by the Netherlands Organisation of Scientific Research (NWO): 016.Veni.192.103. This work was carried out on the Dutch national e-infrastructure with the support of SURF Cooperative. Supported by EPSRC projects EP/K034413/1, EP/K011715/1, EP/L00058X/1, EP/N027833/1, EP/N028201/1, EP/T006544/1.
